# Implementation of a Virtual Point-of-Care Ultrasound Curriculum at Black Lion Hospital, Ethiopia

**DOI:** 10.7759/cureus.68545

**Published:** 2024-09-03

**Authors:** Krisha Desai, Hannibal Kassahun, Abdurezak Ahmed, Dawit K Huluka, Hanan Yusuf, Paulina A Rebolledo, Russell R Kempker, Manpreet Malik

**Affiliations:** 1 Department of Medicine, Emory University School of Medicine, Atlanta, USA; 2 Department of Internal Medicine, Addis Ababa University, Addis Ababa, ETH

**Keywords:** department of medicine, medical education, global health, point-of-care ultrasound, virtual workshop

## Abstract

Introduction

In low- and middle-income countries (LMICs), the availability of formal point-of-care ultrasound (POCUS) training remains limited, and there is limited data on how to train providers in these countries to use POCUS. This study aimed to describe a virtual training workshop for physicians in Ethiopia, with the intention of serving as a model that could guide similar initiatives.

Methods

The authors developed and implemented a three-day virtual workshop in 2022 for physicians in the Department of Medicine at Addis Ababa University in Ethiopia. Participants in the workshop completed pre-workshop and post-workshop surveys, as well as knowledge assessments. The authors examined POCUS use prior to the workshop, the impact of the workshop on ratings of comfort level in performing POCUS, and the change in scores on a knowledge assessment before and after the workshop.

Results

We found that very few of the participants had prior formal POCUS training. Participants reported a significantly higher level of comfort in using POCUS for the assessment of patients (p < 0.001) and for procedural guidance (p < 0.001) after attending the workshop, compared to before. Scores on the post-test knowledge assessment were significantly higher than scores on the pre-test knowledge assessment (p < 0.001).

Conclusion

Our POCUS workshop was successfully implemented and delivered virtually to a group of physicians in Ethiopia, and it increased comfort levels in performing POCUS and POCUS knowledge. We hope that similar workshops can be implemented in other LMICs.

## Introduction

Point-of-care ultrasound (POCUS) is increasingly being recognized as an important skill among internal medicine residency programs. Ultrasound can be used to supplement physical exam findings, improve diagnostic accuracy, and reduce complications for invasive bedside procedures. The implementation of a formal ultrasound curriculum for trainees can also impact patient management [1,2].

POCUS is a modality that can serve as an additional tool for diagnosis and procedural assistance in low- and middle-income countries (LMICs), and there have been prior global health initiatives to increase access to POCUS in resource-limited settings [3]. Particularly because traditional radiologic modalities can require expensive equipment, maintenance, and trained specialists to read images, training clinicians in POCUS can reduce the need for more expensive radiologic tests, using more affordable portable hand-held ultrasound systems [4,5].

A few studies have developed guides to implementing POCUS curricula in other countries, and these training programs generally include a needs assessment, development of objectives and core competencies based on the needs assessment, and post-training assessment of the trainees’ skills [4-6]. One of the primary challenges in implementing POCUS in LMICs is the difficulty of providing consistent, longitudinal training due to geographic distance and logistical feasibility constraints, which may be addressed by virtual POCUS training initiatives. We are not aware of any studies that describe a virtual POCUS curriculum in an LMIC. We aimed to describe the development and feasibility of implementing a virtual three-day POCUS workshop for physicians at Addis Ababa University (AAU) School of Medicine in Ethiopia, with the hope that it can serve as a guide for implementing similar workshops in other LMICs.

## Materials and methods

Study design and participant selection

A study team comprising an Emory University internal medicine resident, an AAU internal medicine chief resident, Emory University Department of Medicine (DOM) faculty physicians, and AAU DOM faculty physicians developed a three-day virtual POCUS workshop. DOM physicians, including faculty, fellows, and internal medicine residents, at AAU were given the opportunity to sign up for the workshop in May 2022, consisting of three one-and-a-half-hour-long sessions broadcast over Zoom™ to a conference room in Black Lion Hospital. Participants in the study completed a pre-workshop survey and knowledge assessment, as well as a post-workshop survey and knowledge assessment. All participants who were physicians at AAU were eligible for inclusion in the study. Prior to the start of the workshop, we purchased a wireless handheld ultrasound for the AAU DOM.

The primary study outcome measure was the impact of the ultrasound workshop on POCUS knowledge. Secondary study outcome measures included the impact of the workshop on confidence levels in performing POCUS and attitudes towards POCUS after completion of the workshop. The study protocol was determined to be exempt from review based on Emory University Institutional Review Board (IRB) guidelines and was approved by the AAU Internal Medicine Department IRB.

Study setting

Ethiopia is a low-income country in the Horn of Africa, a region of eastern Africa. AAU is a national university located in Addis Ababa, Ethiopia, and Black Lion (Tikur Anbessa) Hospital is the main teaching hospital of the university and the largest referral center in the country, where specialized clinical services are available that are not readily accessible in other institutions in Ethiopia. The DOM, including the internal medicine residency program, has limited access to bedside ultrasound, and there has been no formal POCUS training available to physicians in the DOM. The workshop was primarily targeted toward internal medicine residents, although all AAU physicians were invited to participate.

Needs assessment

To design the workshop, we began with a needs assessment as a guide to developing a tailored, locally relevant curriculum, which allowed us to identify the clinical and educational requirements of the participants. A needs assessment is crucial for POCUS training in LMICs because it can help identify and prioritize the specific clinical needs unique to these settings and enhance understanding of the local healthcare context, available resources, and the target population’s experience with ultrasound. We drew on existing literature on prior POCUS curricula and guidelines for conducting a needs assessment in resource-limited settings to develop ours, which comprised a pre-workshop survey, pre-workshop knowledge assessment, structured interviews with AAU leadership, and group discussions with AAU faculty members and residents [4,5,7,8].

Both the pre-workshop survey and knowledge assessment were electronically distributed. The pre-workshop survey (Supplemental Appendix 1) was developed using examples of similar surveys in the literature, and, in combination with the group discussions, the survey helped determine provider-centered needs for the course by assessing the participants’ experience with ultrasound, perceived barriers to learning and using ultrasound, time and financial resources, desire for ultrasound training, and interest in specific POCUS applications [4,7,9-11]. The survey additionally included Likert scale questions about attitudes towards POCUS prior to the workshop. The pre-workshop knowledge assessment consisted of multiple-choice questions used to assess the participants’ baseline POCUS knowledge and to inform the curriculum content and POCUS application focus.

The structured interviews with leadership in the DOM were designed to elicit detailed information on hospital resources and the logistical challenges of implementing the course, and, by engaging department leaders, we ensured that the curriculum was aligned with institutional priorities and had the necessary support for successful implementation. As part of the needs assessment, we also had group discussions, including both AAU faculty and residents, that enabled a multifaceted exploration of needs, expectations, and potential challenges associated with ultrasound training by determining language preferences, patient population, local burden of disease, and how POCUS would intersect with local capacity and resources. We learned through our discussions that AAU DOM did not have a functioning ultrasound machine, which led us to purchase a handheld ultrasound device for the department. Our selection of the device was driven by supporting literature and practical advantages in resource-limited settings, including portability, affordability, and its ability to operate without a stable power supply; we also ensured that technical support would be available to the device users if there was a malfunction [4,12].

A proposal for the POCUS workshop, crafted based on the needs assessment, was disseminated to AAU leadership, faculty members, and internal medicine chief residents, for gathering further input and ensuring the proposal matched identified needs and available resources.

Curriculum development

To develop the curriculum for our POCUS workshop, we conducted an extensive review of the literature concerning the utilization of ultrasound in LMICs, the implementation of POCUS curricula in resource-limited settings, and a standardized diagnostic ultrasound curriculum as proposed by the WHO. In combination with our needs assessment, the literature review provided insights that guided content creation, allowing us to focus on ultrasound topics that offer the greatest utility in the local context.

Common POCUS applications in LMICs in Africa include cardiac ultrasound for the recognition of pericardial effusions and evaluation of cardiac function, lung ultrasound for undifferentiated dyspnea, abdominal ultrasound for the evaluation of free fluid and hydronephrosis, and vascular ultrasound for the diagnosis of deep venous thrombosis (DVT). Our POCUS workshop included education on these topics as well as general POCUS principles [11,13-16]. The workshop used a slideshow format organized by organ system, including cardiac, lung, abdominal, and vascular ultrasound. For each system, we incorporated POCUS videos and case-based learning, enabling participants to practice ultrasound interpretation and apply their ultrasound knowledge to real-life clinical scenarios. The presentations were all designed to allow participants to actively participate, ask questions, and discuss cases in depth. While virtual training has limitations compared to in-person workshops, we aimed to provide an experience that was as close to practical, hands-on learning as possible by using an interactive case-based approach and real-time POCUS video analysis.

Although there is limited data on the use of procedural ultrasound in LMICs, we also incorporated training on procedural ultrasound, recognizing its established role in high-income countries (HICs) as a standard of care for various procedures, including central line placement, thoracentesis, and paracentesis. Multiple studies have shown that ultrasound guidance can improve patient safety, decrease the number of attempts, and reduce healthcare costs [17-19]. Based on our group discussions and survey, the use of ultrasound for these procedures was not routine at Black Lion Hospital.

The workshop was delivered over three consecutive days in May 2022. Each day's session lasted one and a half hours and was broadcast via Zoom™. This virtual delivery model not only facilitated broad accessibility but also allowed for dynamic interaction between participants and instructors. An Emory internal medicine resident led the sessions, with contributions from both Emory and AAU faculty members experienced in POCUS. The workshop's agenda and specific POCUS applications included in the curriculum are detailed in Table 1 of our manuscript. This table serves as a concise guide to the scope and structure of the training provided, illustrating how the sessions were organized to cover a broad range of topics relevant to the needs of healthcare providers in LMICs. By aligning the workshop content with the most pressing clinical needs and available resources, we aimed to maximize the impact of our training on the quality of patient care in these settings.

**Table 1:** POCUS workshop agenda POCUS: Point-of-care ultrasound; BLUE: Bedside lung ultrasound in emergency; IVC: Inferior vena cava; RV: Right ventricle

Day 1 (Session 1)	Day 2 (Session 2)	Day 3 (Session 3)
Basics of ultrasound	Lung ultrasound	Vascular ultrasound
Physics of ultrasound	Basic sonographic windows (including BLUE protocol)	Differentiating veins and arteries
Probe selection and orientation	Pneumothorax	Assessment of deep venous thrombosis
Ultrasound settings	Pleural effusion	IVC ultrasound
Cardiac ultrasound	B-Lines and signs of consolidation	Procedural ultrasound
Basic sonographic windows	Abdominal ultrasound	Central line: placement and prevention of complications
Pulmonary embolism with RV dysfunction	Abdominal free fluid	Paracentesis: placement and assessing complications
Pericardial effusion and cardiac tamponade	Hydronephrosis	Thoracentesis: placement and assessing complications
Assessment of systolic function	Bladder volume	Lumbar puncture: placement
Evaluation of rhythm during cardiopulmonary resuscitation	-	-
Valvular vegetations	-	-

Assessment

We asked participants to complete a post-workshop survey and knowledge assessment within two weeks of completing the workshop. The post-workshop survey (Supplemental Appendix 2) included Likert scale questions about attitudes towards POCUS after completion of the workshop, in addition to open-ended questions soliciting feedback regarding the workshop. The post-workshop knowledge assessment, similar to the pre-workshop knowledge assessment, included multiple-choice questions to assess POCUS knowledge after completion of the workshop. Although no standardized POCUS test exists, we developed a non-validated assessment to gauge POCUS knowledge, modeled after questions from prior POCUS tests found in the literature. Our questions were designed to evaluate core competencies integral to established POCUS curricula and those recommended for POCUS training in LMICs, which include mainly cardiac, pulmonary, and abdominal applications [5,10,20]. The post-test consisted of 15 questions, predominantly focused on POCUS video interpretation, and tested on topics including general ultrasound principles, identification of specific cardiac structures, diagnosis of pleural and pericardial effusions, recognition of pneumothorax, assessment of right heart dysfunction in suspected pulmonary embolism, inferior vena cava (IVC) assessment, and DVT evaluation.

We awarded certificates to participants who fulfilled the following requirements: (I) completed the pre-workshop survey and assessment, (II) attended two out of three sessions in the workshop, and (III) completed the post-workshop survey and assessment.

Data collection and statistical analysis

Data were collected from the online pre- and post-workshop surveys and knowledge assessments. All survey results and assessment scores were de-identified and stored in a computer database for analysis. Data were analyzed using Microsoft Excel 16.54 for Mac (Microsoft® Corp., Redmond, WA, USA) and IBM SPSS Statistics for Windows, Version 29 (Released 2023; IBM Corp., Armonk, NY, USA). Descriptive analyses were carried out for the categorical variables. Differences in survey responses and scores on the knowledge assessment before and after the workshop were compared using a two-sample t-test for data that were normally distributed. The Kolmogorov-Smirnov test was used to test for normality.

## Results

A total of 57 participants completed the pre-workshop survey and knowledge assessment. The demographics of the participants are shown in Table 2. The majority were male (n = 35, or 61.4%) and aged between 26 and 35 years old (n = 54, or 94.7%). Nearly all participants were internal medicine residents (n = 54, or 94.7%), with a similar distribution among year 1 (n = 20, or 35.1%), year 2 (n = 20, or 35.1%), and year 3 (n = 14, or 24.6%) residents. A total of 45 participants completed the post-workshop survey and knowledge assessment; the majority were male (n = 25, or 55.6%), aged between 26 and 35 years old (n = 42, or 93.3%), and internal medicine residents (n = 43, or 95.6%). All participants who completed the post-workshop survey and knowledge assessment attended at least two sessions, with 17.7% attending two sessions (n = 8) and 82.2% attending all three sessions (n = 37).

**Table 2:** Demographics of participants in virtual POCUS workshop at Black Lion Hospital POCUS: Point-of-care ultrasound

Demographics	Pre-workshop survey, N = 57 (%)	Post-workshop survey, N = 45 (%)
Gender		
Female	22 (38.6)	20 (44.4)
Age		
16-25	1 (1.8)	1 (2.2)
26-35	54 (94.7)	42 (93.3)
36-45	2 (3.5)	2 (4.4)
Level of training		
Residency	54 (94.7)	43 (95.6)
Year 1	20 (35.1)	14 (31.1)
Year 2	20 (35.1)	16 (35.6)
Year 3	14 (24.6)	13 (28.9)
Chief resident	1 (1.8)	1 (2.2)
Fellow	1 (1.8)	0 (0)
Faculty member	1 (1.8)	1 (2.2)

Table 3 demonstrates the use of ultrasound by the participants before completing the workshop. While many had previously performed POCUS (n = 39, or 68.4%), the majority had not received formal ultrasound training (n = 56, or 98.2%). Most had performed fewer than 20 scans previously (n = 39, or 68.4%), and only six participants (10.6%) reported performing POCUS more than five times a month. More than half of the participants had previously performed lung ultrasound (n = 34, or 59.6%), with volume assessment (n = 28, or 49.1%) and cardiac ultrasound (n = 25, or 43.9%) as the next most frequently performed exams. While approximately a third of participants had never performed an ultrasound-guided procedure (n = 21, or 36.8%), a substantial proportion had previously performed ultrasound-guided thoracentesis (n = 29, or 50.9%), paracentesis (n = 17, or 29.8%), and vascular access procedures (n = 12, or 21.1%).

**Table 3:** POCUS use by participants prior to completing the virtual POCUS workshop ^a^Vascular access included placement of central venous catheters and peripheral venous catheters. POCUS: Point-of-care ultrasound; FAST: Focused assessment with sonography for trauma

	N = 57 (%)
Prior use of POCUS	
Yes	39 (68.4)
No	18 (31.6)
Number of total POCUS scans performed	
0	18 (31.6)
1-10	11 (19.3)
11-20	10 (17.5)
21-40	9 (15.8)
>40	9 (15.8)
Frequency of performing POCUS	
Never	18 (31.6)
<1 time a month	12 (21.5)
1-5 times a month	21 (36.7)
6-15 times a month	5 (8.8)
>15 times a month	1 (1.8)
POCUS exams previously performed	
None	18 (31.6)
Cardiac/Echocardiography	25 (43.9)
Lung	34 (59.6)
Abdominal	15 (26.3)
FAST	15 (26.3)
Volume assessment	28 (49.1)
Ultrasound-guided procedures previously performed	
Never	21 (36.8)
Paracentesis	17 (29.8)
Thoracentesis	29 (50.9)
Pericardiocentesis	2 (3.5)
Vascular access^a^	12 (21.1)
Joint aspiration	2 (3.5)
Lumbar puncture	0 (0)
Prior formal ultrasound training	
Yes	1 (1.8)
No	56 (98.2)

**Figure 1:**
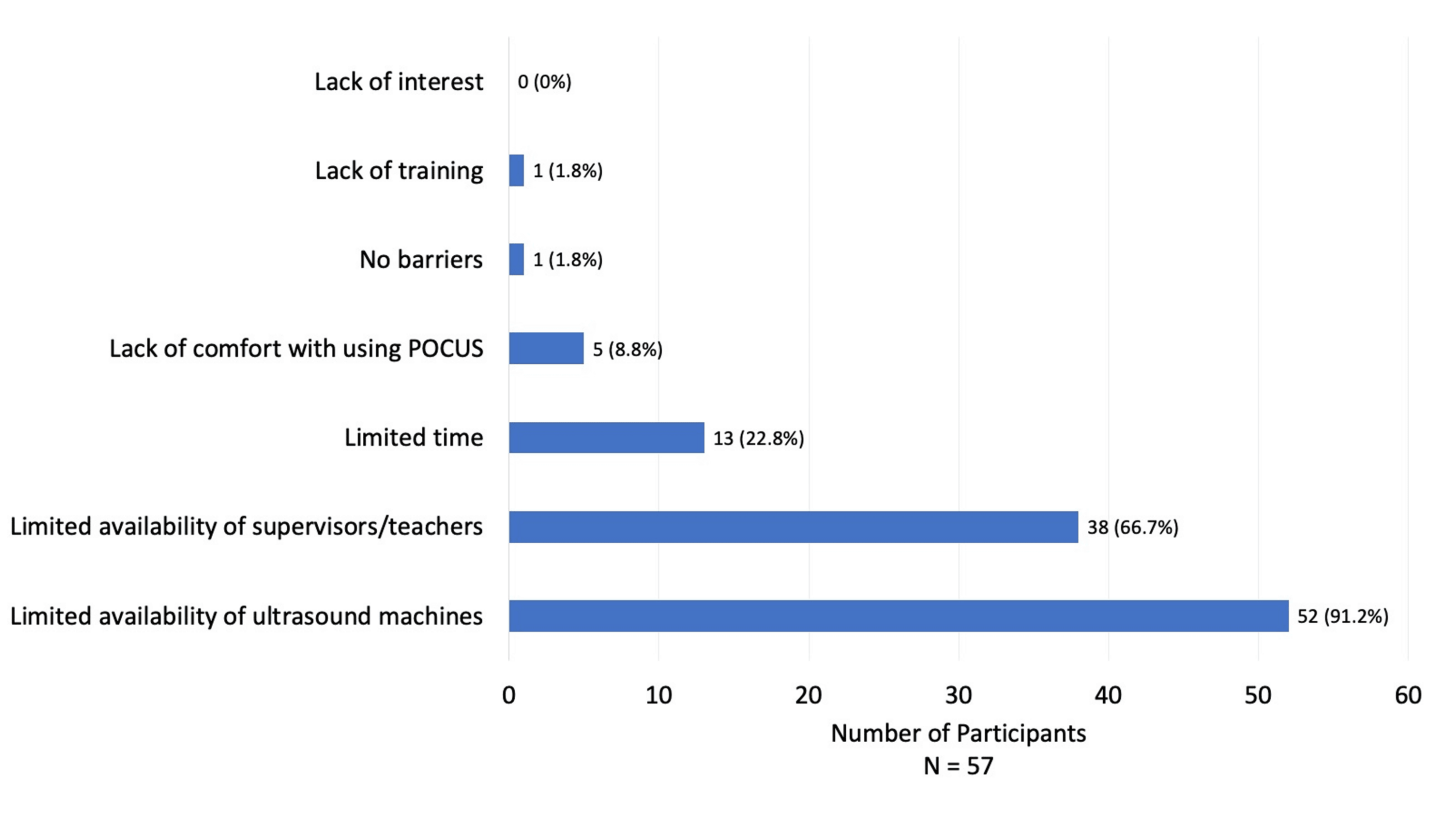
Barriers to point-of-care ultrasound (POCUS) use The graph illustrates the perceived barriers to POCUS use by physicians at Black Lion Hospital.

Figure 1 demonstrates perceived barriers to POCUS use by the participants, with the most reported barrier being limited availability of ultrasound machines (n = 52, or 91.2%), followed by limited availability of supervisors/teachers (n = 38, or 66.7%), limited time (n = 13, or 22.8%), and lack of comfort with using POCUS (n = 5, or 8.8%). Very few participants reported lack of training (n = 1, or 1.8%) as a barrier, and no participants cited lack of interest (0%) as a barrier. Only one participant (1.8%) reported no barriers to POCUS use.

Table 4 shows mean ratings on Likert scale questions asked before and after the workshop, with a five-point Likert scale used (1 = Strongly Disagree, 2 = Disagree, 3 = Neutral, 4 = Agree, 5 = Strongly Agree). When comparing responses after the workshop to those before, participants reported a significantly higher level of comfort in using POCUS for the assessment of patients (mean rating 4.5 (SD = 0.6) vs. 3.6 (SD = 1.3), p < 0.001) and for procedural guidance (mean rating 4.4 (SD = 0.4) vs. 3.4 (SD = 1.4), p < 0.001) after the workshop. On the post-workshop compared to the pre-workshop survey, there was no statistically significant difference in the participants’ interest in additional POCUS training (mean rating 4.8 (SD = 0.6) vs. 4.9 (SD = 0.3), p = 0.06). When asked whether the workshop increased their confidence in performing and interpreting POCUS, the mean rating was 4.5 (SD = 0.6). When participants were asked if they would use POCUS more for the assessment of patients and procedural guidance after completing the workshop, the mean ratings were 4.9 (SD = 0.3) and 4.8 (SD = 0.5), respectively.

**Table 4:** Virtual POCUS participant responses to Likert scale questions on pre-workshop and post-workshop surveys ^a^A 5-point Likert scale was used as follows: 1 = Strongly Disagree, 2 = Disagree, 3 = Neutral, 4 = Agree, 5 = Strongly Agree. POCUS: Point-of-care ultrasound

Likert scale questions^a^	Pre-workshop survey mean rating (SD)	Post-workshop survey mean rating (SD)	p-value
“I am comfortable using point-of-care ultrasound for the assessment of patients”	3.6 (1.3)	4.5 (0.6)	p < 0.001
“I am comfortable using point-of-care ultrasound for procedural guidance”	3.4 (1.4)	4.4 (0.7)	p < 0.001
“I am interested in additional training in point-of-care ultrasound”	4.9 (0.3)	4.8 (0.6)	p = 0.06
“The POCUS workshop increased my confidence level in performing and interpreting point-of-care ultrasound.”	-	4.5 (0.6)	-
“If available, I will use POCUS more often for assessment of patients after completion of the workshop.”	-	4.9 (0.3)	-
“If available, I will use POCUS more often for procedural guidance after completion of the workshop.”	-	4.8 (0.5)	-

**Figure 2:**
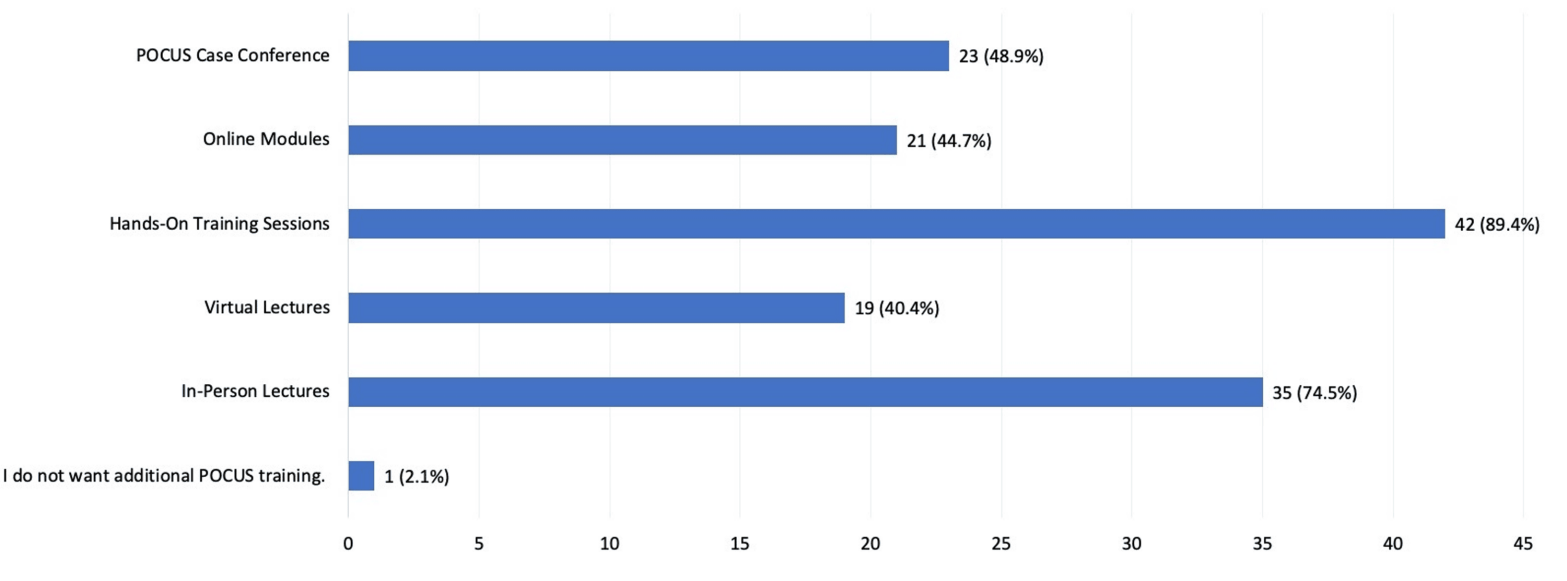
Interest in additional point-of-care ultrasound (POCUS) training This graph shows the types of additional POCUS training that participants were interested in receiving after completion of the virtual POCUS workshop at Black Lion Hospital.

After completion of the workshop, a significant proportion of the participants reported interest in additional hands-on training sessions (n = 42, or 89.4%), followed by in-person lectures (n = 35, or 74.5%), as demonstrated in Figure 2. Only one participant (2.1%) reported not wanting additional POCUS training.

The average score on the post-test knowledge assessment (72.4%, SD = 2.2%) was significantly higher than the average score on the pre-test knowledge assessment (57.9%, SD = 1.6%; p < 0.001).

## Discussion

This study describes the development and implementation of a three-day POCUS workshop for DOM physicians in Addis Ababa, Ethiopia. POCUS has shown increasing promise in LMICs as a modality that can effectively guide the diagnosis and management of patients, and more affordable portable handheld ultrasound devices can replace expensive radiologic tests [4,12-14,21]. The COVID-19 pandemic necessitated the virtual format of the workshop, and, to our knowledge, no other virtual POCUS workshops delivered to an LMIC have been described in the literature. The historical challenges of providing continuous and high-fidelity POCUS training in LMICs - often exacerbated by the transient nature of traditional in-person teaching - are significant, and virtual training has the potential to mitigate these challenges by enabling more consistent and accessible education, thus better supporting local communities’ capacity to utilize POCUS effectively.

Similar to this study, limited POCUS training has been reported in other LMICs [11,22]. Physicians in HICs, including internal medicine physicians, typically report a higher frequency of ultrasound use compared to the participants in our workshop, more than half of whom reported using ultrasound less than once a month [23,24]. This disparity could be a result of our study participants mostly being internal medicine residents, but there are very few studies that report on ultrasound use by exclusively internal medicine residents. For the participants who had performed POCUS before the workshop, the most common diagnostic exams were lung, cardiac, and volume assessments. Prior studies evaluating ultrasound use in LMICs have reported that abdominal ultrasound, particularly in the setting of trauma (i.e., focused assessment with sonography for trauma (FAST)), obstetrics, and cardiac ultrasound were the most common diagnostic ultrasound types, although most of these studies were done in emergency departments [13,16]. While there is a paucity of data on POCUS use by DOM physicians in LMICs, a large survey of hospitalists in the U.S. reported that the most commonly performed diagnostic exams were cardiac and lung ultrasound, similar to the participants in this study, suggesting that many of the POCUS applications used by DOM physicians in HICs can be incorporated in LMICs [25]. Despite ultrasound guidance being a standard of care for procedures such as paracentesis, thoracentesis, and central line placement in HICs, more than a third of the survey participants had not performed an ultrasound-guided procedure, which may be attributed to a lack of access to POCUS training and/or absence of locally adapted guidelines.

The most commonly reported barrier to POCUS use was the limited availability of POCUS devices, which is in accordance with the results of prior surveys conducted in LMICs, where the top barriers to ultrasound use were around access (e.g., access to machines, access to maintenance of machines, and cost of ultrasound machines) [11,22,26]. Providing LMICs with increased access to devices, as we have done with a portable handheld ultrasound device, may, in turn, facilitate ultrasound use. The barriers next most reported by the AAU physicians were limited availability of supervisors/teachers, limited time, and lack of comfort with POCUS - all of which were common barriers reported in surveys performed in both LMICs and HICs. Although lack of training was also a barrier often reported in these prior surveys, less than 2% of the AAU survey participants reported this as a barrier [11,22,25].

While longitudinal POCUS training is the recommended model for POCUS education, short POCUS workshops have been shown to increase confidence and comfort levels with performing POCUS, improve POCUS knowledge, and increase bedside ultrasound utilization [5,9,11,27,28]. Although our POCUS workshop was virtual and did not include hands-on POCUS training like many of the prior short workshops described in the literature, it resulted in improved comfort levels in using POCUS for the assessment of patients and for procedural guidance. Participants reported high levels of confidence in performing and interpreting POCUS after the workshop, indicating that a short, virtual workshop may facilitate ultrasound use in LMICs, as lack of comfort is a common barrier to use. Our results also show that the workshop increased POCUS knowledge in the short term, as evidenced by the pre-test and post-test scores.

Strengths of the study include feasibility, ease of implementation, and relatively low resource utilization, as the only major cost was the purchase of the ultrasound device. Since Likert scale questions are a universal method for survey collection, our surveys can be readily adapted in other institutions, and the pre-test and post-test designs are an efficient way of measuring changes due to a specific intervention. This study also had limitations. While we conducted the survey and post-test immediately after the completion of the workshop, we did not evaluate the longer-term durability of the workshop on comfort level, POCUS knowledge, or ultrasound use. We also acknowledge that heightened confidence or comfort with POCUS tools does not necessarily equate to improved competency - a phenomenon known as the Dunning-Kruger effect, which is well-documented in medical education research, including specific studies on POCUS [29,30]. In addition, our short-term knowledge assessment focuses on immediate recall and may not reflect the ability to apply knowledge in clinical settings, depth of understanding, or decision-making skills required for effective POCUS application. Future studies should therefore include performance-based assessments to more accurately evaluate the impact of our training interventions. Since longitudinal curricula are important for sustainable POCUS education and retention of POCUS skills and knowledge, we are currently developing a longitudinal component to the training program, including hands-on training in Ethiopia by Emory University faculty trained in POCUS [4,5].

## Conclusions

We successfully developed and delivered a three-day virtual POCUS workshop for physicians in Ethiopia, highlighting the feasibility of such training. Prior to the workshop, many participants had no formal POCUS training and limited POCUS usage, emphasizing the need for this program. To address the main barrier - lack of access to equipment - we provided a portable handheld ultrasound. The workshop not only increased comfort with POCUS for both diagnostic and procedural uses but also improved knowledge. Future studies should focus on evaluating the competency in ultrasound that participants achieve following ultrasound training, as well as assessing the longitudinal impact of such a training program in other geographic areas and patient populations.

## Appendices

Supplemental appendix 1

Pre-Workshop Survey

(1) What gender do you identify as?

A. Male

B. Female

C. Other

D. Prefer not to answer

(2) What is your age?

A. 0 - 15 years old

B. 16 - 25 years old

C. 26 - 35 years old

D. 36 - 45 years old

E. 46 - 55 years old

F. Over 55 years old

(3) What is your current level of training?

A. Medical Student

B. 1st Year Resident

C. 2nd Year Resident

D. 3rd Year Resident

E. Chief Resident

F. Fellow

G. Faculty Member

H. Other: _________________

(4) Have you ever used an ultrasound machine for point-of-care ultrasound?

A. Yes

B. No

(5) If you have used an ultrasound machine before, approximately how many ultrasounds have you performed overall during your medical career (including medical school and residency)?

A. 0

B. 1-10

C. 11-20

D. 2-40

E. >40

(6) On average, how often do you currently perform point-of-care ultrasound?

A. >15 times in one month

B. 6-15 times in one month

C. 1-5 times in one month

D. <1 time a month

E. Never

(7) Please check all the types of point-of-care ultrasound exams you have performed:

A. Cardiac/Echocardiography

B. Lung

C. Abdominal (liver, gallbladder, spleen, kidneys, etc.)

D. Focused Assessment with Sonography for Trauma (FAST)

E. Volume assessment

F. Procedural

G. I have not performed a point-of-care ultrasound exam.

H. Other: _________________

(8) If you have performed ultrasound-guided procedures, for which of the following procedures have you used ultrasound? Check all that apply.

A. Paracentesis

B. Thoracentesis

C. Pericardiocentesis

D. Vascular Access (central lines, vascular catheters, etc.)

E. Joint Aspiration

F. Lumbar Puncture

G. I have not performed ultrasound-guided procedures.

H. Other: _________________

(9) Have you received any formal ultrasound training during residency thus far?

A. Yes

B. No

(10) Which of the following are barriers to using point-of-care ultrasound? Check all that apply.

A. Limited time

B. Availability of ultrasound machines

C. Availability of supervisors/teachers

D. Lack of comfort with using POCUS

E. Lack of interest

F. I believe POCUS has limited value

F. Other: _________________

G. No barriers

(11) Which POCUS topics do you feel would be most useful? Check all that apply.

A. Cardiac/Echocardiography

B. Lung

C. Abdominal (liver, gallbladder, spleen, kidneys, etc.)

D. FAST/Trauma

E. Volume assessment

F. Procedural

G. Other: _________________

For the following questions, rate your level of agreement with each statement.

(12) Point-of-care ultrasound is a useful adjunct to the traditional physical examination.

1: Strongly Disagree

2: Disagree

3: Neutral

4: Agree

5: Strongly Agree

(13) I am comfortable using point-of-care ultrasound for the assessment of patients.

1: Strongly Disagree

2: Disagree

3: Neutral

4: Agree

5: Strongly Agree

(14) I am comfortable using point-of-care ultrasound for procedural guidance.

1: Strongly Disagree

2: Disagree

3: Neutral

4: Agree

5: Strongly Agree

(15) Increased access to ultrasound equipment will increase my use of point-of-care ultrasound.

1: Strongly Disagree

2: Disagree

3: Neutral

4: Agree

5: Strongly Agree

(16) I am interested in additional training in point-of-care ultrasound.

1: Strongly Disagree

2: Disagree

3: Neutral

4: Agree

5: Strongly Agree

(17) Increased training in point-of-care ultrasound will increase my confidence level in performing and interpreting point-of-care ultrasound.

1: Strongly Disagree

2: Disagree

3: Neutral

4: Agree

5: Strongly Agree

(18) Confidence in performing and interpreting point-of-care ultrasound will result in healthcare cost savings.

1: Strongly Disagree

2: Disagree

3: Neutral

4: Agree

5: Strongly Agree

(19) Confidence in performing and interpreting point-of-care ultrasound will enhance my diagnostic abilities.

1: Strongly Disagree

2: Disagree

3: Neutral

4: Agree

5: Strongly Agree

(20) Confidence in performing and interpreting point-of-care ultrasound will help me in the management of my patients.

1: Strongly Disagree

2: Disagree

3: Neutral

4: Agree

5: Strongly Agree

Supplemental appendix 2

Post-Workshop Survey

(1) Which of the following POCUS sessions did you attend? Check all that apply.

A. Session 1 (May 24)

B. Session 2 (May 25)

C. Session 3 (May 26)

D. I did not attend any of the sessions.

For questions (2)-(10), rate your level of agreement with each statement.

(2) The POCUS workshop increased my confidence level in performing and interpreting point-of-care ultrasound.

1: Strongly Disagree

2: Disagree

3: Neutral

4: Agree

5: Strongly Agree

(3) If available, I will use POCUS more often for the assessment of patients after the completion of the workshop.

1: Strongly Disagree

2: Disagree

3: Neutral

4: Agree

5: Strongly Agree

(4) If available, I will use POCUS more often for procedural guidance after completion of the workshop.

1: Strongly Disagree

2: Disagree

3: Neutral

4: Agree

5: Strongly Agree

(5) I am comfortable using point-of-care ultrasound for the assessment of patients.

1: Strongly Disagree

2: Disagree

3: Neutral

4: Agree

5: Strongly Agree

(6) I am comfortable using point-of-care ultrasound for procedural guidance.

1: Strongly Disagree

2: Disagree

3: Neutral

4: Agree

5: Strongly Agree

(7) Overall, the POCUS workshop was well-organized.

1: Strongly Disagree

2: Disagree

3: Neutral

4: Agree

5: Strongly Agree

(8) The pace of the POCUS workshop was appropriate.

1: Strongly Disagree

2: Disagree

3: Neutral

4: Agree

5: Strongly Agree

(9) The POCUS workshop was too advanced for my level of knowledge.

1: Strongly Disagree

2: Disagree

3: Neutral

4: Agree

5: Strongly Agree

(10) I am interested in additional training in point-of-care ultrasound.

1: Strongly Disagree

2: Disagree

3: Neutral

4: Agree

5: Strongly Agree

(11) What additional POCUS training would you be interested in receiving? Check all that apply.

A. In-person lectures

B. Virtual lectures

C. Hands-on training sessions

D. Online, self-paced modules

E. POCUS case conference

F. Other: _______________

For the questions (12)-(16), rate how helpful the following topics in the POCUS workshop were:

(12) Cardiac POCUS:

1: Not at all helpful

2: Mostly not helpful

3: Neutral

4. Somewhat helpful

5. Extremely helpful

(13) Lung POCUS:

1: Not at all helpful

2: Mostly not helpful

3: Neutral

4. Somewhat helpful

5. Extremely helpful

(14) Abdominal (liver, gallbladder, spleen, kidneys, etc.) ultrasound:

1: Not at all helpful

2: Mostly not helpful

3: Neutral

4. Somewhat helpful

5. Extremely helpful

(15) Volume Assessment:

1: Not at all helpful

2: Mostly not helpful

3: Neutral

4. Somewhat helpful

5. Extremely helpful

(16) Procedural ultrasound:

1: Not at all helpful

2: Mostly not helpful

3: Neutral

4. Somewhat helpful

5. Extremely helpful

For the following questions, please evaluate the instructors (participants were asked the following three questions for each instructor).

(17) Overall, this instructor was an effective teacher.

1: Strongly Disagree

2: Disagree

3: Neutral

4: Agree

5: Strongly Agree

(18) The instructor clearly presented explanations of important concepts.

1: Strongly Disagree

2: Disagree

3: Neutral

4: Agree

5: Strongly Agree

(19) The instructor created an environment that was respectful and inclusive of students’ diverse backgrounds and ideas.

1: Strongly Disagree

2: Disagree

3: Neutral

4: Agree

5: Strongly Agree

Open-ended questions:

(20) What was most helpful about this POCUS workshop?

(21) What did you like least about this POCUS workshop?

(22) What specific suggestions do you have about improving the POCUS workshop?

(23) Please use this space for any additional comments, questions, or suggestions regarding the course.
